# Integrated Computational Investigation of *Cannabis sativa* Phytoconstituents as Putative Multi-Target Inhibitors in Skin Cancer: A Molecular Docking, Dynamics, and ADMET Profiling Study

**DOI:** 10.3390/ph19020315

**Published:** 2026-02-13

**Authors:** Lamiae El Bouamri, Salma Laaouina, Ibtissam Lakrim, Hassan Nour, Imane Yamari, Abdelouahid Samadi, Mohammed Bouachrine, Samir Chtita

**Affiliations:** 1 Laboratory of Analytical and Molecular Chemistry, Faculty of Sciences Ben M’Sick, Hassan II University of Casablanca, Casablanca 20670, Morocco; elbouamrilamiae14@gmail.com (L.E.B.); slaaouinasalma@gmail.com (S.L.); lakrimibtissam5@gmail.com (I.L.); hassannour737@gmail.com (H.N.); yamariimane86@gmail.com (I.Y.); 2 Department of Chemistry, College of Science, United Arab Emirates University, Al Ain P.O. Box 15551, United Arab Emirates; 3 MCNS Laboratory, Faculty of Sciences, Moulay Ismail University, Meknes 50000, Morocco; bouachrine@gmail.com

**Keywords:** *Cannabis sativa*, skin cancer, EGFR, BRAF V600E, TGF-β, molecular docking, molecular dynamics simulations, MM-GBSA, ADMET prediction

## Abstract

**Background**: Skin cancer progression is driven by the dysregulation of key oncogenic signaling pathways, including EGFR, BRAF V600E, and TGF-β, which collectively promote tumor proliferation, invasion, and metastatic progression. Targeting these pathways using multitarget natural modulators represents a promising therapeutic strategy. **Methods**: In this study, forty-nine phytoconstituents from *Cannabis sativa* were evaluated using an integrated computational approach to explore their inhibitory potential against EGFR, BRAF V600E, and the TGF-β receptor. Molecular docking was performed to assess binding affinities and interaction profiles, followed by ADMET analysis to evaluate pharmacokinetic and safety properties. The top-ranked compounds were further investigated using 200 ns molecular dynamics simulations and MM-GBSA binding free energy calculations to assess the stability and strength of protein–ligand interactions. **Results**: Several phytoconstituents exhibited strong binding affinities toward the target proteins, formed stable interactions with key active-site residues, and demonstrated favorable pharmacokinetic profiles with acceptable safety characteristics. Molecular dynamics simulations confirmed the structural stability of the selected protein–ligand complexes, while MM-GBSA analysis supported their favorable binding energetics. **Conclusions**: These findings suggest that *Cannabis sativa* phytoconstituents may represent a promising source of multitarget modulators capable of attenuating EGFR, BRAF V600E, and TGF-β driven oncogenic signaling in skin cancer. This study provides a mechanistic framework that supports further in vitro validation and the development of cannabis-derived therapeutic candidates for targeted skin cancer management.

## 1. Introduction

*Cannabis sativa*, belonging to the Cannabaceae family, is a multipurpose plant widely recognized for its broad spectrum of pharmacological activities that include anti-inflammatory effects [1], antioxidant effects [2], neuroprotective effects [3], and anxiolytic effects [4]. Such biological properties are widely associated with its rich composition of bioactive phytochemicals, notably cannabinoids [5], terpenes [6], and flavonoids [7], which, through interaction with the endocannabinoid system, modulate important physiological processes [8]. Beyond its classic use in traditional medicine, such bioactive molecules are increasingly attracting the attention of cosmetic science due to their capacity to reduce cutaneous inflammation [9], regulate sebum secretion [10], counteract skin oxidative stress [11], and improve skin regeneration [12]. After the medical and cosmetic use of Cannabis was legalized by Moroccan Law [13,14,15,16,17,18,19,20,21], interest in *Cannabis sativa*-derived molecules for dermotherapeutic formulations has become a promising area of scientific and industrial innovation [13]. More than 550 bioactive compounds have been identified within C. sativa, making it a rich pharmacological plant worthy of interest [14]. Its components, especially cannabinoids, are highly valued for their potential role in modulating cellular signaling pathways related to inflammation [15], oxidative stress [16], and uncontrolled cell proliferation [17], all critical processes in oncogenesis and skin aging [18]. Basal cell carcinoma, squamous cell carcinoma, and melanoma are major cutaneous oncological diseases with a profound impact on world health [19]. Basal and squamous cell carcinomas are the most common [20], whereas melanoma remains the most aggressive due to its high metastatic potential [21].

The pathophysiology of these malignancies is closely associated with the dysregulation of critical signaling cascades such as epidermal growth factor receptor (EGFR) signaling [22], BRAF V600E mutation-driven MAPK pathway activation [23], and aberrant transforming growth factor-β (TGF-β) signaling [24]. Together, these pathways orchestrate abnormal cell proliferation, survival, angiogenesis, invasion, immune evasion, and therapeutic resistance [25]. Targeting these molecular determinants, therefore, represents a compelling strategy for both skin cancer therapy [26] and anti-aging interventions [27]. Notably, several phytoconstituents of *Cannabis sativa* have demonstrated the ability to interact with these oncogenic pathways, positioning them as dual-action agents that combine dermocosmetic benefits with anticancer potential [28]. In this context, the present study aims to identify and characterize bioactive phytoconstituents of *Cannabis sativa* that may exhibit inhibitory activity against key molecular targets involved in skin cancer development, namely EGFR, BRAF V600E, and TGF-β [29]. Molecular docking, molecular dynamics (MD) simulations, and MM-PBSA binding free energy estimations were performed, together with ADMET profiling of pharmacokinetic and toxicity parameters [30]. The objective is to discover novel candidates with favorable energetic profiles and drug-likeness properties through the screening of a curated library of forty-nine phytochemicals derived from *Cannabis sativa* [31]. Beyond skin cancer, the top-scoring Cannabis-derived phytochemicals may exert beneficial effects in other pathological contexts due to their potential polypharmacological properties. These include anti-inflammatory and antioxidant activities, modulation of fibrotic processes via TGF-β, neuroprotective effects through EGFR, BRAF V600E and TGF-β signaling, and regulation of metabolic pathways. Notably, these pathways are implicated in various cancers, including colorectal and lung cancer, as well as systemic diseases characterized by chronic inflammation and fibrosis, highlighting the broader therapeutic potential of these phytocompounds [32]. This work provides a robust theoretical framework for the rational development of cannabis-based dermotherapeutic agents capable of modulating oncogenic signaling in skin carcinogenesis and contributing to innovative strategies for skin health and cancer prevention.

## 2. Results

### 2.1. Docking Outcomes

A total of forty-nine cannabinoids and cannabis-derived phytoconstituents were computationally screened to evaluate their therapeutic potential against skin cancer. Molecular docking simulations were carried out to investigate the binding performance of these molecules within the active sites of the three major oncogenic targets involved in skin tumor initiation and progression, namely BRAF V600E, EGFR, and TGF-β [33]. The obtained docking binding affinities for all screened phytoconstituents are reported in Table 1, highlighting the most promising cannabis compounds able to interact strongly with these cancer-related proteins.

Molecular docking simulations were performed to evaluate the binding affinity of forty-nine *Cannabis sativa*-derived phytoconstituents toward the three selected oncogenic targets, namely EGFR, BRAF V600E, and TGF-β [34]. The obtained docking binding free energies provide a quantitative assessment of the interaction strength between each ligand and the corresponding protein active site. Table 1 summarizes the binding free energies (ΔG, kcal/mol) of all screened cannabinoids and terpenoids, allowing a comparative analysis of their predicted inhibitory potential against these cancer-related proteins.

The docking analysis, performed for forty-nine *Cannabis sativa*-derived phytoconstituents against EGFR, BRAF V600E, and TGF-β, showed that the binding free energies were negative for all compounds interacting with the active sites of these oncogenic targets. As summarized in Table 1, several phytoconstituents showed higher binding affinities compared to the reference drugs Erlotinib (EGFR, −7.5 kcal/mol), Vemurafenib (BRAF V600E, −6.5 kcal/mol), and Galunisertib (TGF-β, −6.1 kcal/mol). Indeed, the highest interactions against all three targets were represented by C5, C6, and C7, with binding energies of −8.5, −9.9, and −9.4 kcal/mol for C5; −8.8, −9.6, and −9.2 kcal/mol for C6; and −9.6, −9.8, and −9.2 kcal/mol for C7 against EGFR, BRAF V600E, and TGF-β, respectively.

Based on the docking results presented in Table 1, the phytoconstituents exhibiting the most favorable binding affinities toward all three targets were further highlighted. the top-ranking compounds (C5–C7), along with their corresponding binding energies against EGFR, BRAF V600E, and TGF-β, in comparison with the reference drug are reports in Table 2. This focused comparison enables a clearer evaluation of the most promising *Cannabis sativa*-derived candidates for further molecular interaction and dynamic analyses [35].

**Figure 1:**
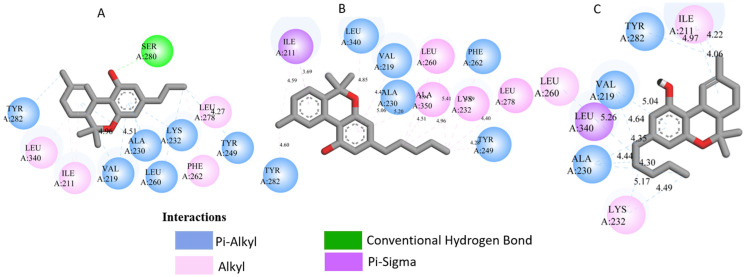
Molecular docking interactions of *Cannabis sativa* phytoconstituents with the TGF-β receptor (PDB ID: 3TZM): (**A**) C5, (**B**) C6, and (**C**) C7.

**Figure 2:**
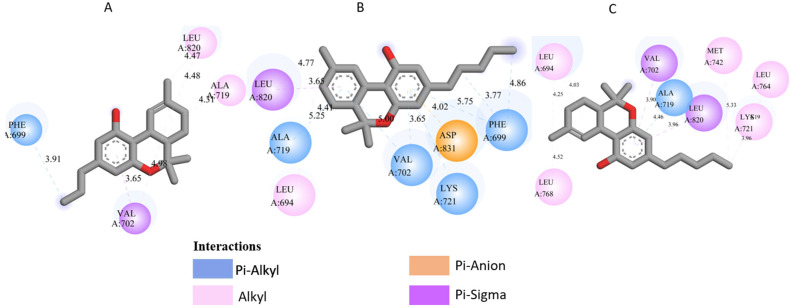
Molecular docking interactions of *Cannabis sativa* phytoconstituents with the EGFR (PDB ID: 1M17): (**A**) C5, (**B**) C6, and (**C**) C7.

**Figure 3:**
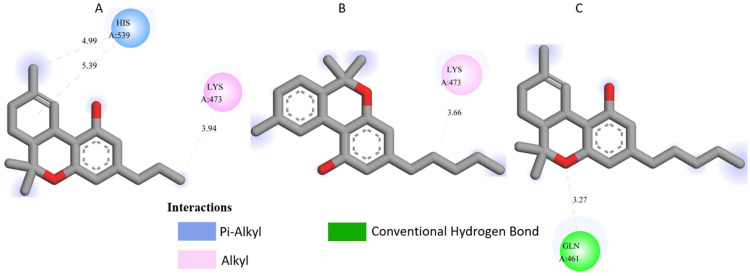
Molecular docking interactions of *Cannabis sativa* phytoconstituents with BRAF V600E (PDB ID: 5RGQ): (**A**) C5, (**B**) C6, and (**C**) C7.

All three *Cannabis sativa* phytoconstituents THCV (C5), CNB (C6), and 9-THC/Dronabinol (C7) exhibited negative binding free energies against EGFR, BRAF V600E, and TGF-β, confirming favorable and spontaneous interactions with the target proteins (Table 2). The molecular interactions between the selected phytoconstituents (C5, C6, and C7) and the target enzymes EGFR, BRAF V600E, and TGF-β are illustrated in Figure 1, Figure 2 and Figure 3. These visualizations highlight key hydrogen bonds, hydrophobic contacts, and π–π stacking interactions that stabilize the ligand–protein complexes, providing a structural basis for their high binding affinities and potential inhibitory activity [36].

### 2.2. Ligand–Target Interaction Analysis

Table 3 presents the detailed interaction profiles of the selected top-ranking phytoconstituents (C5, C6, and C7) with the target proteins 3TZM (TGF-β), 1M17 (EGFR), and 5JRQ (BRAF V600E). The table summarizes the key residues involved in ligand binding, the types of interactions (including conventional hydrogen bonds, π–π stacking, π–alkyl, π–cation, and hydrophobic contacts), and the corresponding distances in angstroms. These interactions provide a structural rationale for the high binding affinities observed in the docking analysis and highlight specific amino acids critical for complex stabilization. Similar interaction patterns have been reported to contribute significantly to the inhibitory activity of bioactive compounds against these oncogenic targets.

Docking analysis was performed for forty-nine *Cannabis sativa*-derived phytoconstituents against EGFR, BRAF V600E, and TGF-β. The binding free energies were negative for all compounds interacting with the active sites of these oncogenic targets. As summarized in Table 1 and illustrated in Figure 1, Figure 2 and Figure 3, several phytoconstituents showed higher binding affinities compared to the reference drugs Erlotinib (EGFR, −7.5 kcal/mol), Vemurafenib (BRAF V600E, −6.5 kcal/mol), and Galunisertib (TGF-β, −6.1 kcal/mol). The compounds with the strongest binding against all three targets were C5, C6, and C7, with binding energies of −8.5, −9.9, and −9.4 kcal/mol for C5; −8.8, −9.6, and −9.2 kcal/mol for C6; and −9.6, −9.8, and −9.2 kcal/mol for C7 against EGFR, BRAF V600E, and TGF-β, respectively. Other compounds, such as C9 and C11, also showed promising docking scores.

Ligand–receptor interaction analyses were further carried out for the selected ligands, as depicted in Figure 1, Figure 2 and Figure 3 and Table 3. The key active site residues of the three target proteins were identified using Discovery Studio based on the observed interactions with the ligands. For 3TZM, residues involved included Ser280, Tyr282, Leu289, Leu340, and Ile211; for 1M17, Phe699, Leu820, Val702, and Ala719; and for 5JRQ, His539 and Lys473. These residues formed conventional hydrogen bonds, π–π stacking, π–alkyl interactions, and hydrophobic contacts with the ligands, with distances ranging from 2.4 to 5.2 Å. These residues were used to define the binding site grid for molecular docking, ensuring reproducibility and accurate ligand positioning.

- 3TZM complexes: All ligands interact with key hydrophobic residues in the binding cavity. C5 forms a hydrogen bond with SER A: 280 and hydrophobic contacts with TYR A: 282, LEU A: 340, and ILE A: 211. C6 is stabilized via hydrophobic and π-alkyl interactions with LEU A: 278, VAL A: 219, ALA A: 230, and TYR A: 249, while C7 shows multiple hydrophobic interactions with VAL A: 219, LEU A: 260, and ALA A: 230 and contact with ILE A: 211.
- 1M17 complexes: C5 formed a π-π interaction with PHE A: 699 and van der Waals contacts with LEU A: 820, and VAL A: 702. C6 is hydrogen-bonded to ASP A: 831 and interacts with PHE A: 699, LEU A: 820, and VAL A: 719. C7 exhibits extensive hydrophobic interactions with ALA A: 719, LEU A: 820, and LYS A: 721.
- 5JRQ complexes: C5 forms weak hydrogen bonds with HIS A: 539 and LYS A: 473, C6 interacts mainly with LYS A: 473, and C7 forms a strong hydrogen bond with GLN A: 461 along with additional nonpolar contacts.

### 2.3. Drug-Likeness, Pharmacokinetics, and Pharmacodynamics Investigation Comprehensive ADMET Profiling of Selected Cannabis sativa Compounds

To assess the suitability of the selected *Cannabis sativa* phytoconstituents (C5, C6, and C7) as potential candidates for topical treatment of skin cancer, key drug-likeness parameters were evaluated, including molecular weight (MW), lipophilicity (LogP), and the number of hydrogen bond acceptors (n-HBA) and donors (n-HBD). All three compounds fully comply with Lipinski’s rule of five: MW ≤ 500, LogP ≤ 5, n-HBA ≤ 10, and n-HBD ≤ 5. These characteristics suggest a favorable balance between hydrophilicity and lipophilicity, supporting effective skin permeability and bioavailability [36]. LogP values ranging from 1.60 to 4.05 indicate adequate lipophilicity, which is crucial for enabling penetration through the lipid-rich layers of the skin. The summary of the ADME-Tox evaluation of the selected *Cannabis sativa* compounds, namely C5, C6, and C7, is shown in Table 4, Table 5 and Table 6. This gives an overview of the pharmacokinetic behavior, dermatological safety, and suitability for topical applications. All three compounds comply with Lipinski’s and Veber’s rules by having molecular weights below 500 g/mol, with moderate to high lipophilicity (LogP 1.60–4.05), limited hydrogen bond donors and acceptors, and optimal rotatable bonds and polar surface area. These features indicate an optimum balance between hydrophilicity and lipophilicity, which is critical for good permeation through the stratum corneum and subsequent bioavailability in the viable epidermis or dermis. High gastrointestinal absorption is predicted for all compounds, reflecting overall good systemic bioavailability; for topical applications, this primarily ensures safety in case of minor systemic exposure [37]. Calculation of skin permeability coefficients (Log Kp between −2.737 and −2.538 cm/s) indicates a moderate transdermal penetration, which allows the delivery of bioactive molecules to the target skin layers without excessive systemic absorption, suggesting that C5-C7 are excellent candidates for dermocosmetic applications, such as antioxidant, anti-inflammatory, or anti-aging formulations. Furthermore, all compounds are not predicted to inhibit any major CYP450 isoforms (CYP1A2, CYP2C19, CYP2C9, CYP2D6, CYP3A4), which reduces the possibility of metabolic interactions, while the toxicity assessment using ProTox-II verified the three compounds as non-hepatotoxic, non-carcinogenic, non-mutagenic, and non-cytotoxic with LD50s from 482 to 13,500 mg/kg (Table 6)**,** supporting a high safety margin for topical use. In addition, the corresponding BBB predictions give low permeation rate values for C5 and C7, along with controlled permeability in the case of C6, thus minimizing the risk of side effects through the CNS [38]. These findings reinforce the pharmacological potency of compounds C5–C7, which may act as active principles of dermocosmetic formulations and local treatments against skin cancer, by combining good local action with acceptable skin permeation and reduced systemic toxicity [39].

### 2.4. Molecular Dynamics Simulations

#### 2.4.1. Molecular Dynamics Simulation of Cannabinoid–Protein Complexes: RMSD, RMSF, Radius of Gyration, SASA, and PSA

**Figure 4:**
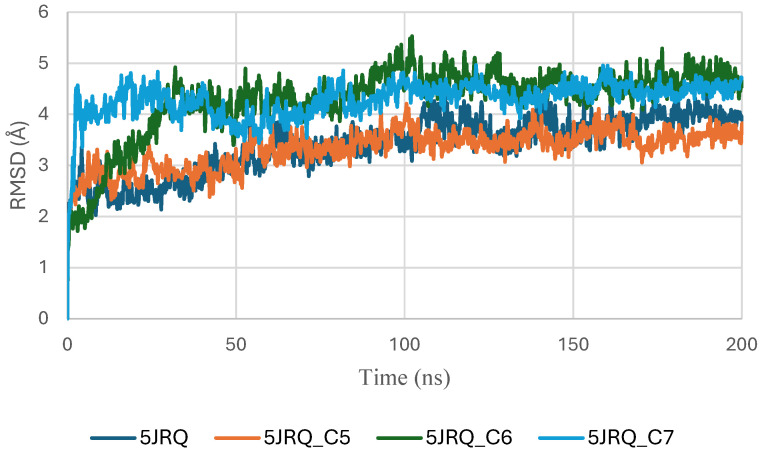
RMSD plot of EGFR (5JRQ) complexes with C5, C6 and C7.

The RMSD trajectories of EGFR (PDB ID: 5JRQ) in both its apo form and in complex with ligands C5, C6, and C7 were analyzed over a 200 ns molecular dynamics simulation to evaluate the structural stability of each system (Figure 4). The apo protein displayed the highest degree of fluctuation, with RMSD values ranging from approximately 3.0 to 4.8 Å, reflecting its intrinsic flexibility and the absence of stabilizing interactions in the binding pocket [40].

In contrast, all ligand-bound complexes exhibited lower and more consistent RMSD values, indicating enhanced structural stabilization upon ligand binding [41]. Among the tested systems, the EGFR–C6 complex demonstrated the most stable profile, reaching equilibrium within the first 20 ns and maintaining a narrow fluctuation range around 2.5–3.2 Å. This behavior suggests a strong anchoring of C6 within the active site and minimal perturbation of the protein backbone. The EGFR–C5 complex showed slightly higher variability, fluctuating between 2.8 and 3.6 Å, yet remained within a stable regime, indicating that C5 provides a stabilizing effect, albeit less pronounced than C6. The EGFR–C7 complex exhibited moderate fluctuations (3.0–4.0 Å), consistent with stable but more dynamic protein–ligand interactions [24].

Overall, the reduced RMSD amplitudes in the ligand-bound systems compared to the apo form confirm that all three phytoconstituents stabilize EGFR throughout the simulation. Among them, C6 appears to provide the highest structural stabilization. These results align with docking and binding energy analyses and further support the potential of C5, C6, and C7 as promising EGFR modulators [42,43].

**Figure 5:**
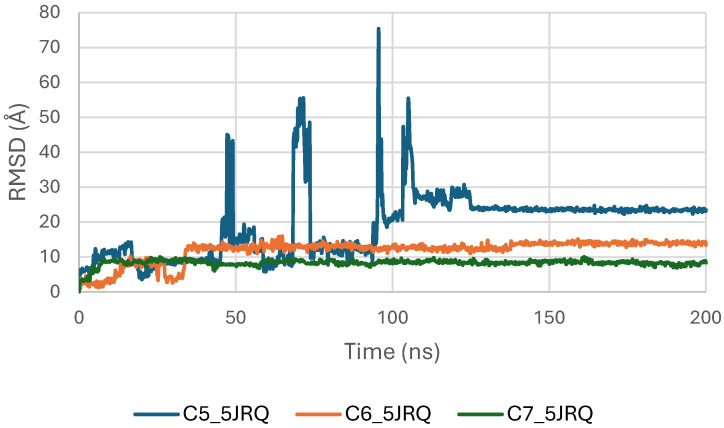
RMSD plot of ligands inside EGFR (5JRQ) binding pocket.

Figure 5 presents the RMSD trajectories of the ligands C5, C6, and C7 within the EGFR (5JRQ) binding pocket over the 200 ns simulation. This analysis provides insight into the conformational stability and retention of each ligand inside the active site throughout the trajectory. All three molecules exhibit an initial equilibration phase during the first few nanoseconds, followed by globally stable RMSD values, indicating that they maintained consistent orientations within the catalytic cavity. Among them, C6 shows the lowest and most stable fluctuations, reflecting strong anchoring and persistent binding interactions. C7 also demonstrates stable behavior with moderate variations, while C5 undergoes slightly higher early fluctuations before stabilizing. The overall stability observed for the three ligands aligns well with their favorable docking affinities and MM-GBSA energies, supporting the formation of robust and persistent ligand–protein complexes.

**Figure 6:**
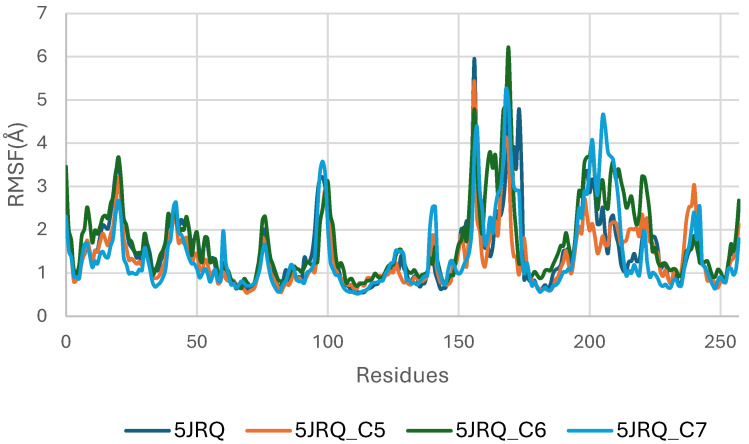
Root Mean Square Fluctuation (RMSF) Profiles of EGFR (5JRQ) Apo and Complexed with C5, C6, and C7.

Figure 6 illustrates the RMSF profiles of EGFR (5JRQ) in its apo form and in complex with the ligands C5, C6, and C7, providing detailed insight into residue-level flexibility across the protein structure. The apo receptor exhibits the highest fluctuation amplitudes, particularly within the loop-rich and solvent-exposed regions, approximately spanning residues 40–70, 120–150, and 190–230. These elevated fluctuations reflect the inherent dynamic nature of unbound EGFR, which undergoes broader conformational motions in the absence of stabilizing ligand interactions. In contrast, all ligand-bound complexes display a general reduction in RMSF values, confirming that ligand binding contributes to dampening local flexibility and enhancing the structural stability of EGFR. Among the complexes, C6 induces the most pronounced stabilization, with markedly lower fluctuations in the vicinity of the active site, consistent with its strong anchoring and favorable binding energetics observed in docking and MM-GBSA analyses. The C5–EGFR and C7–EGFR systems also show reduced flexibility relative to the apo state, although moderate fluctuations persist in certain regions, particularly around residues 180–210, suggesting localized motions that do not compromise overall structural integrity. Importantly, none of the ligand-bound systems exhibit excessive fluctuations in the catalytic domain, confirming that ligand binding preserves the functional architecture of EGFR. Collectively, the RMSF results indicate that C5, C6, and C7 stabilize the receptor to varying extents, with C6 providing the most significant dampening of dynamic fluctuations, in agreement with RMSD and binding free energy evaluations.

**Figure 7:**
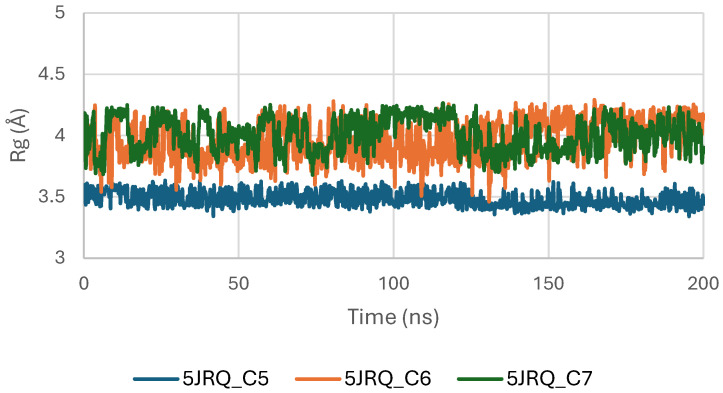
Radius of Gyration (Rg) of EGFR (5JRQ) complexes.

Figure 7 presents the Radius of Gyration (Rg) profiles of EGFR (5JRQ) in complex with the ligands C5, C6, and C7 throughout the 200 ns molecular dynamics simulation. The Rg values remain highly stable for all systems, fluctuating within a narrow window of approximately 3.8–4.2 Å, which indicates that the global compactness of the protein is well preserved during the simulation. This consistent behavior confirms the absence of major structural expansion or collapse, reflecting stable tertiary architecture under ligand-bound conditions.

Among the analyzed complexes, the C6–EGFR system displays the lowest and most uniform Rg values, suggesting a slightly more compact and stabilized conformation compared with the C5- and C7-bound forms. The C5–EGFR and C7–EGFR complexes also maintain regular and controlled fluctuations, showing no significant deviations that would indicate destabilization or unfolding events. The overall stability of the Rg trajectories indicates that ligand binding does not disrupt the structural integrity of EGFR. Instead, the stable compactness patterns reaffirm that C5, C6, and C7 establish energetically favorable and structurally compatible interactions with the receptor, with C6 inducing the highest degree of conformational tightening. These findings are consistent with the RMSD and MM-GBSA analyses, which also identified C6 as the most stabilizing ligand.

**Figure 8:**
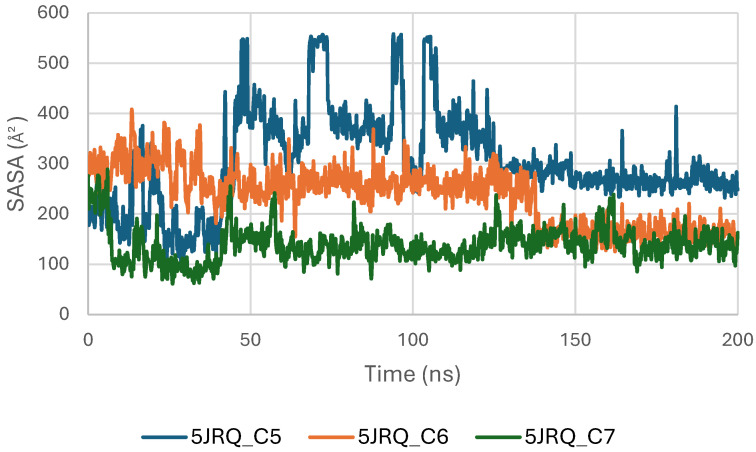
Solvent Accessible Surface Area (SASA) of EGFR (5JRQ) complexes.

Figure 8 presents the Solvent Accessible Surface Area (SASA) profiles of EGFR (5JRQ) in complex with ligands C5, C6, and C7 over a 200 ns molecular dynamics simulation. The SASA trajectories of all systems remain within a stable range of approximately 250–450 Å^2^, indicating that EGFR does not undergo significant structural expansion or collapse during ligand binding. This overall stability reflects the preservation of the protein’s solvent exposure and surface topology throughout the simulation.

Among the three complexes, the C6–EGFR system exhibits the most stable SASA values, with minimal fluctuations, suggesting that C6 binding maintains a compact and well-organized protein surface. The C7 complex follows a similarly consistent profile, with moderate but controlled variations, confirming that no major perturbations occur in the solvent-exposed regions of the receptor. The C5–EGFR complex displays slightly higher SASA oscillations, particularly during the first 50 ns, corresponding to early structural adjustments before reaching a stable equilibrium phase.

Overall, the SASA analysis confirms that ligands C5, C6, and C7 do not induce destabilizing conformational changes in EGFR. The stability of SASA across all systems supports the structural integrity of the receptor during ligand binding, with C6 providing the most compact and stabilized complex, in agreement with RMSD, Rg, and MM-GBSA observations.

**Figure 9:**
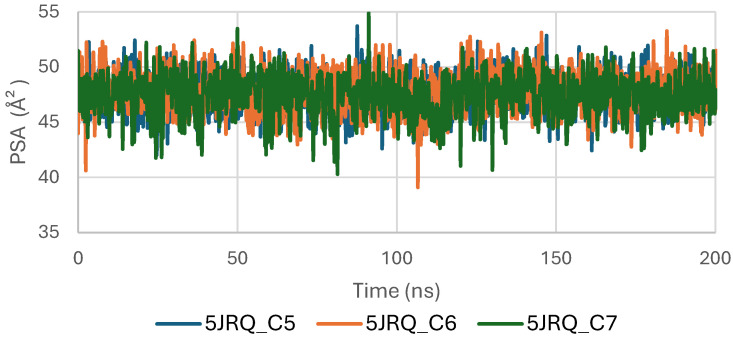
Polar Surface Area (PSA) evolution of EGFR (5JRQ) complexes.

Figure 9 shows the Polar Surface Area (PSA) evolution of EGFR (5JRQ) in complex with ligands C5, C6, and C7 over a 200 ns molecular dynamics simulation. All systems exhibit PSA values fluctuating within a narrow and stable range of approximately 35–45 Å^2^, with no abrupt transitions or major deviations. This consistent behavior indicates that the polar regions of EGFR exposed to the solvent remain largely unchanged during ligand binding, suggesting that the ligands do not induce significant rearrangements within the protein’s polar surface.

The stability of the PSA trajectories further reflects a well-preserved hydrogen-bonding environment and sustained polar interactions within the binding pocket. Among the complexes, C6 and C7 present slightly smoother curves, indicating marginally higher conformational consistency compared with C5, although all three remain within a similar stability interval.

Overall, the PSA analysis confirms that EGFR maintains a stable polar surface architecture upon interaction with C5, C6, and C7. These results are consistent with the RMSD, RMSF, Rg, and SASA observations, further supporting the formation of structurally stable and dynamically persistent complexes between EGFR and the three cannabinoids.

**Figure 10:**
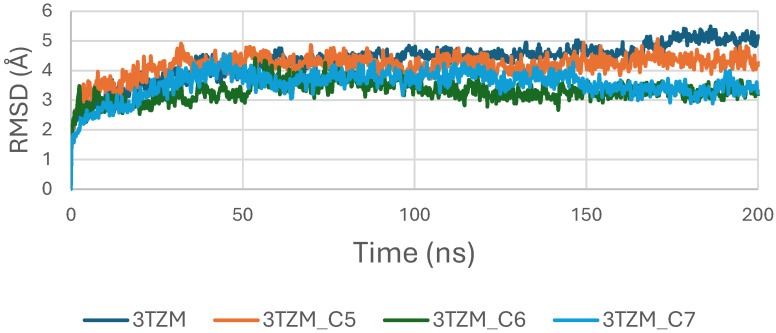
RMSD plot of TGF-β (3TZM) complexes with C5, C6 and C7.

The RMSD trajectories of the TGF-β receptor (3TZM) complexed with ligands C5, C6, and C7 were monitored over 200 ns to assess backbone stability. As shown in Figure 10, all systems exhibit a short equilibration phase during the first ~10–15 ns, corresponding to initial relaxation of the protein–ligand conformations. Following this adjustment, each complex stabilizes within a fluctuation window of ~3.5–4.5 Å.

Among the three systems, the C6–3TZM and C7–3TZM complexes display the smoothest RMSD profiles, with fewer deviations and more uniform trajectories, indicating a more consistent conformational behavior over time. The C5–3TZM complex shows slightly higher fluctuations during the mid-simulation interval, but the RMSD values remain within an acceptable and stable range, suggesting that ligand accommodation does not disrupt the backbone architecture.

Overall, the RMSD curves indicate that all three cannabinoids maintain the receptor in a stable structural state throughout the simulation period.

**Figure 11:**
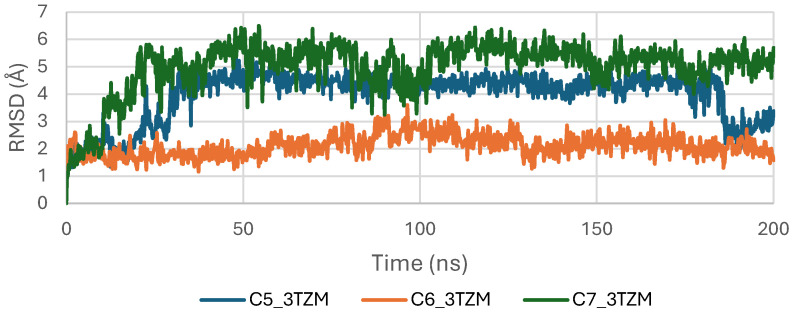
RMSD plot of ligands inside TGF-β (3TZM) binding pocket.

The RMSD trajectories of ligands C5, C6, and C7 inside the binding pocket of the TGF-β receptor (3TZM) were analyzed over the 200 ns simulation to evaluate their conformational stability and retention within the active site. As shown in Figure 11, all ligands exhibit an initial equilibration phase during the first 10–20 ns, corresponding to their adjustment from the docked pose to a more relaxed configuration within the pocket. After equilibration, the three ligands maintain RMSD values that remain predominantly within the 2.5–4.5 Å window, indicating stable accommodation inside the binding site without signs of displacement or detachment [44]. Among them, C6 shows the most stable trajectory, characterized by a smooth and consistently low RMSD profile, suggesting a well-maintained binding orientation throughout the simulation. Ligands C5 and C7 show slightly broader fluctuations, with occasional increases in the mid-simulation segment, yet their RMSD values remain within acceptable stability limits. These RMSD patterns reflect persistent ligand anchoring and limited conformational drift, indicating that C5, C6, and C7 retain favorable orientations inside the TGF-β binding cavity across the entire simulation time [45].

**Figure 12:**
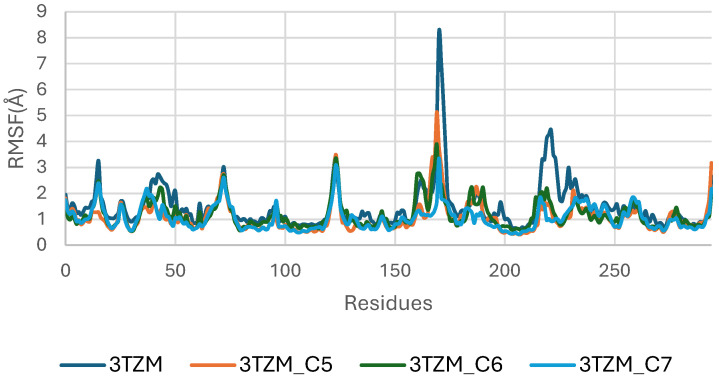
Root Mean Square Fluctuation (RMSF) Profiles of TGF-β (3TZM) Apo and Complexed with C5, C6, and C7.

The RMSF analysis of the TGF-β receptor (3TZM) in its apo form and when bound to ligands C5, C6, and C7 highlights residue-level flexibility across the 200 ns simulation. As shown in Figure 12, most residues exhibit low RMSF values (<3 Å), indicating that the overall backbone dynamics of 3TZM remain stable in all systems.

The apo protein displays higher fluctuations across several loop regions, particularly within residues ~150–170 and ~230–250, which correspond to solvent-exposed and intrinsically flexible segments. Upon ligand binding, these fluctuations are noticeably reduced, especially in the C6- and C7-bound complexes, reflecting a stabilizing influence of the ligands on these dynamic regions.

The C6 complex shows the smoothest fluctuation profile, with consistently dampened mobility across the receptor, suggesting tighter and more persistent interactions at the binding site. The C5 and C7 complexes display slightly higher flexibility in isolated loop segments but remain well within acceptable fluctuation ranges [46].

**Figure 13:**
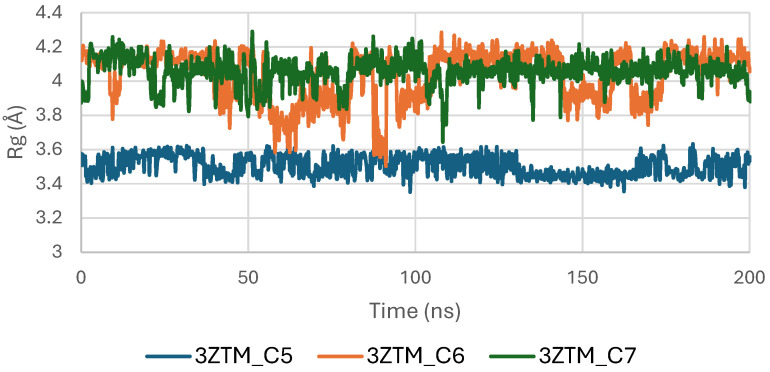
Radius of Gyration (Rg) of TGF-β (3TZM) complexes.

The Radius of Gyration (Rg) of the TGF-β receptor (3TZM) in complexes with ligands C5, C6, and C7 shows a stable pattern throughout the 200 ns simulation. As illustrated in Figure 13, all systems maintain Rg values within a narrow interval of approximately 19.0–19.5 Å, reflecting sustained compactness of the protein’s tertiary structure.

The C6-bound complex presents the smoothest and most uniform Rg trajectory, suggesting slightly enhanced structural stabilization compared with the C5 and C7 complexes. Minor fluctuations observed in the C5 and C7 systems remain within normal dynamic ranges and do not indicate any substantial alteration of the protein’s global fold [47].

Overall, the Rg profiles confirm that ligand binding does not induce significant expansion or contraction of the TGF-β receptor, and the protein maintains consistent compactness across all three complexes.

**Figure 14:**
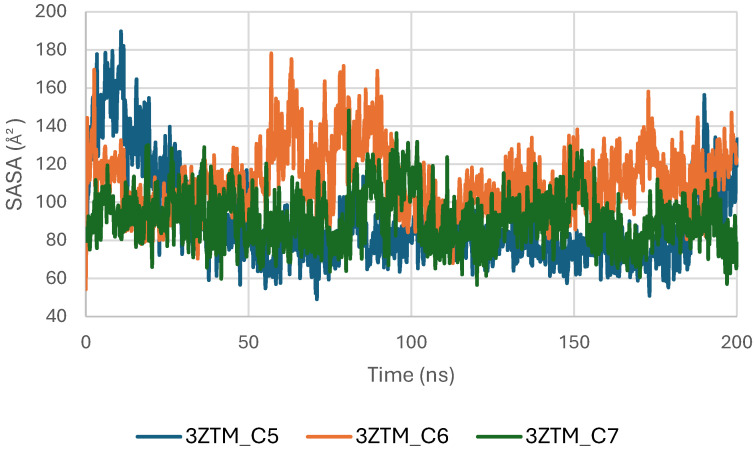
Solvent Accessible Surface Area (SASA) of TGF-β (3TZM) complexes.

The Solvent-Accessible Surface Area (SASA) of the TGF-β receptor (3TZM) was evaluated to assess solvent exposure and potential conformational adjustments upon ligand binding. As shown in Figure 14, the SASA profiles of the complexes with C5, C6, and C7 fluctuate within a moderate and stable range of approximately 90–160 Å^2^ throughout the 200 ns simulation. These variations reflect normal dynamic behavior of surface-exposed regions and do not indicate structural instability.

During the initial phase of the simulation (0–30 ns), slightly higher fluctuations are observed, particularly for the C5 and C6 complexes, corresponding to early relaxation and adjustment of the protein–ligand systems. Beyond this period, the SASA curves stabilize, indicating that each complex reaches a consistent dynamic equilibrium [48].

Among the three systems, the C7 complex shows the most uniform and compact SASA trajectory, suggesting reduced solvent exposure of the protein surface. The C6 system exhibits slightly broader fluctuations, while C5 follows an intermediate profile, but all remain within acceptable ranges for a well-folded protein under physiological conditions.

**Figure 15:**
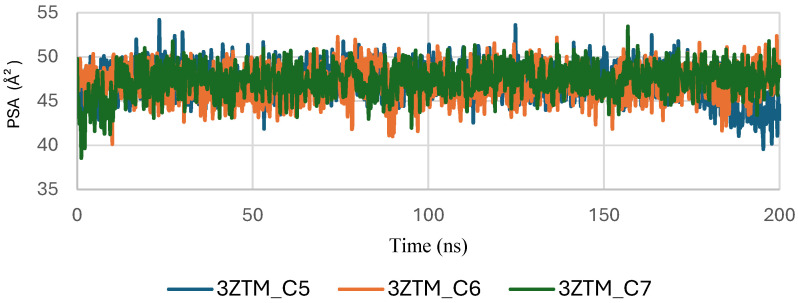
Polar Surface Area (PSA) evolution of TGF-β (3TZM) complexes.

The Polar Surface Area (PSA) analysis of the TGF-β receptor (3TZM) in complexes with ligands C5, C6, and C7 provides insights into the behavior of solvent-exposed polar regions during the 200 ns molecular dynamics simulation. As shown in Figure 15, the PSA values of all three systems fluctuate within a narrow range of approximately 40–55 Å^2^, indicating that the polar surface remains structurally stable throughout the simulation.

A brief adjustment phase is observed during the initial 20–30 ns, corresponding to relaxation of the protein–ligand complexes from their starting conformations. After equilibration, the PSA trajectories show consistent patterns with no abrupt deviations, suggesting that ligand binding does not induce significant changes in the exposure of polar residues.

Among the complexes, C6 and C7 exhibit slightly smoother PSA curves, while C5 displays minor additional fluctuations but remains within the same stability interval. Overall, the stable PSA evolution confirms that the polar regions of TGF-β maintain steady solvent accessibility across all ligand-bound conditions.

**Figure 16:**
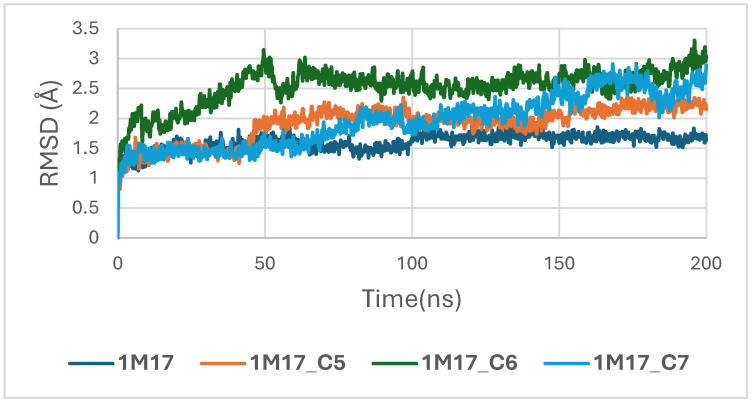
RMSD plot of BRAF (1M17) complexes with C5, C6 and C7.

In the case of the 1M17 protein, the RMSD trajectories of the BRAF kinase (PDB ID: 1M17) in its apo state and when bound to ligands C5, C6, and C7 were analyzed over a 200 ns molecular dynamics simulation to evaluate global structural stability. As shown in Figure 16, the apo-BRAF system stabilizes rapidly after the initial 10–15 ns, maintaining RMSD values between 1.5 and 2.0 Å, which reflects the natural flexibility of the kinase while preserving its overall structural architecture. All ligand-bound complexes exhibit similarly stable RMSD profiles, with fluctuations generally ranging between 1.5 and 3.0 Å throughout the simulation. The C5–BRAF complex displays the narrowest RMSD range, fluctuating between approximately 1.5 and 2.5 Å, indicating that ligand C5 contributes to a well-maintained and tightly stabilized protein backbone. The C7 complex shows comparable behavior, reaching equilibrium early and remaining within a stable interval of 2.0–2.5 Å. In contrast, the C6-bound system presents slightly higher fluctuations, particularly during the 50–120 ns interval, with RMSD values approaching ~3.0 Å; however, these variations remain within acceptable dynamic limits and do not suggest any structural disruption [49].

**Figure 17:**
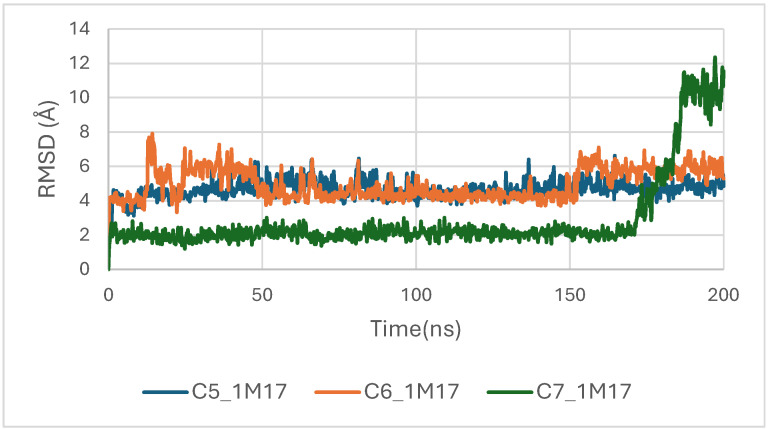
RMSD plot of ligands inside BRAF (1M17) binding pocket.

**Figure 18:**
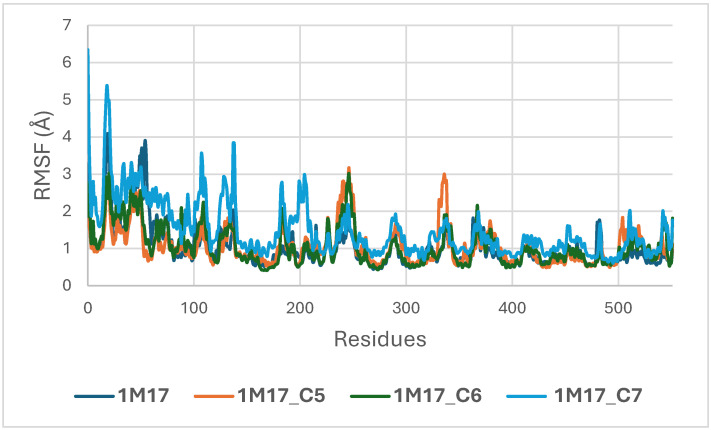
Root Mean Square Fluctuation (RMSF) Profiles of BRAF (1M17) Apo and Complexed with C5, C6, and C7.

**Figure 19:**
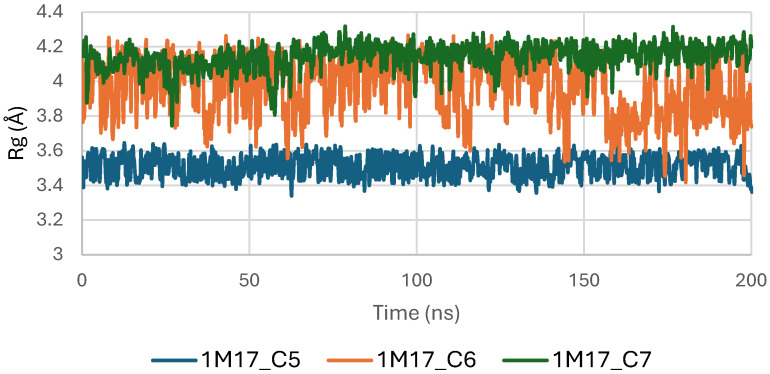
Radius of Gyration (Rg) of BRAF (1M17) complexes.

**Figure 20:**
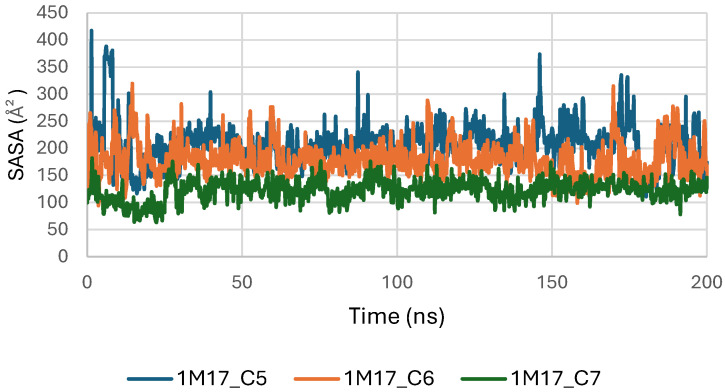
Solvent Accessible Surface Area (SASA) of BRAF (1M17) complexes.

**Figure 21:**
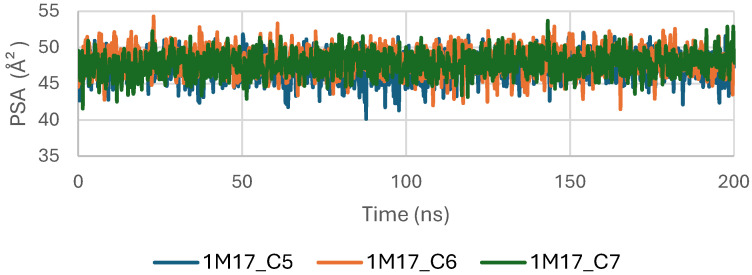
Polar Surface Area (PSA) evolution of BRAF (1M17) complexes.

Concomitantly, the ligand RMSD trajectories (Figure 17) show that C7 preserved a relatively stable binding mode, oscillating within ~2.0–4.0 Å, whereas C5 and C6 exhibited broader excursions (up to ~5–7 Å), yet remained largely confined within the binding site [49]. The RMSF analysis is presented in Figure 18, showing that residue-wise fluctuations were moderate; the highest flexibility was observed in loop regions, while residues forming the active site and the binding pocket remained relatively rigid. This proves that ligand binding does not compromise local structural stability, with the protein maintaining its functional conformation during the course of this simulation [50]. These facts related to global structural compactness were further supported by the values of the Radius of Gyration Rg (Figure 19), which consistently oscillated around ~3.5–4.0 Å, indicating that 1M17 protein maintained a folded state without any denaturation or global expansion upon ligand accommodation. Solvent exposure descriptors further support such behavior. In more detail, SASA evolution (Figure 20) remained globally stable in the range of ~150–250 Å^2^, confirming that the protein–solvent interface is preserved and that ligand binding did not provoke any remarkable hydration rearrangement. Similarly, PSA trajectories (Figure 21) maintained a narrow and steady profile around ~46–58 Å^2^, indicating that no major rearrangement of polar patches occurred during the 100 ns simulation. In summary, these MD indicators confirm the stability over time of the C5–C6–C7 interaction with the 1M17 protein. No significant structural deviation, compaction loss, or hydration perturbation has been detected; thus, these three cannabinoids interact coherently within the 1M17 protein active region during the 100 ns simulation [51].

Figure 16 depicts the root-mean-square deviation (RMSD) of BRAF (1M17) complexes with the top-ranking phytoconstituents C5, C6, and C7 over the course of the molecular dynamics simulations. This analysis evaluates the structural stability and conformational fluctuations of each ligand–protein complex. The RMSD trajectories indicate that all three complexes reached equilibrium and remained relatively stable throughout the simulation, reflecting strong and stable interactions within the active site. These findings are consistent with the high docking scores obtained for these compounds and suggest potential inhibitory activity against BRAF V600E.

Figure 17 presents the RMSD trajectories of the ligands C5, C6, and C7 within the BRAF (1M17) binding pocket during the molecular dynamics simulations. This analysis provides insight into the stability and conformational behavior of each ligand while bound to the active site. The relatively low and stable RMSD values indicate that all ligands maintained consistent orientations within the pocket throughout the simulation, reflecting stable ligand–protein interactions. These findings corroborate the docking results and suggest potential inhibitory activity of the phytoconstituents against BRAF V600E.

Figure 18 illustrates the root-mean-square fluctuation (RMSF) profiles of BRAF V600E (1M17) in its apo form and when complexed with the ligands C5, C6, and C7. RMSF analysis evaluates the flexibility of individual amino acid residues during the molecular dynamics simulations. The plot shows that most residues, particularly those in the active site, exhibit low fluctuations upon ligand binding, indicating that complex formation stabilizes key regions of BRAF V600E. These observations are consistent with the RMSD analysis and support the formation of stable ligand–protein complexes, highlighting the potential inhibitory activity of these phytoconstituents [52].

Figure 19 shows the radius of gyration (Rg) profiles of BRAF V600E (1M17) complexes with ligands C5, C6, and C7. Rg analysis provides insight into the overall compactness and folding stability of the protein during the molecular dynamics simulations. The Rg values remained relatively constant, indicating that ligand binding did not induce significant conformational changes and that BRAF V600E maintained structural integrity.

Figure 20 illustrates the solvent-accessible surface area (SASA) of BRAF (1M17) complexes with ligands C5, C6, and C7 throughout the simulations. SASA analysis reflects the exposure of protein surfaces to the solvent and can indicate conformational changes upon ligand binding. The SASA values remained stable for all complexes, suggesting that ligand binding did not significantly alter the overall surface accessibility and supporting the formation of stable protein–ligand interactions.

Figure 21 shows the evolution of the polar surface area (PSA) of BRAF V600E (1M17) complexes with ligands C5, C6, and C7 during the molecular dynamics simulations. PSA analysis highlights the polar regions of the protein accessible to the solvent, which are important for hydrogen bonding and ligand recognition. The PSA values remained relatively constant throughout the simulation, indicating that the polar surface of BRAF V600E was preserved and that ligand binding maintained structural stability.

#### 2.4.2. Hydrogen Bond (H-Bond) Analysis

**Figure 22:**
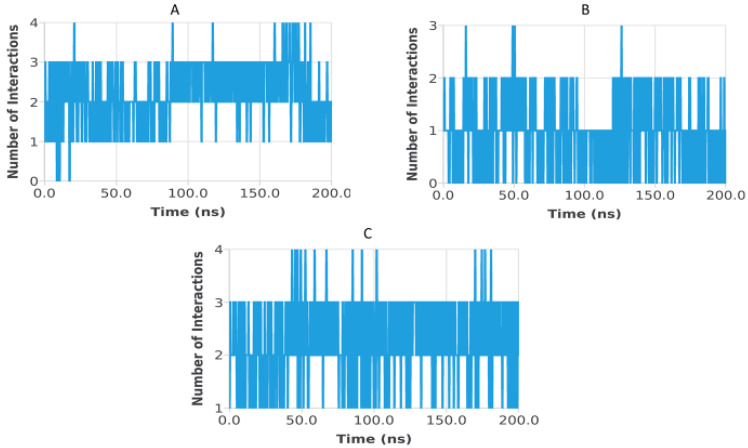
Hydrogen bond evolution of TGF-β receptor (3TZM) complexes with ligands C5, C6, and C7 over 200 ns. (**A**) 3TZM–C5, (**B**) 3TZM–C6, (**C**) 3TZM–C7.

**Figure 23:**
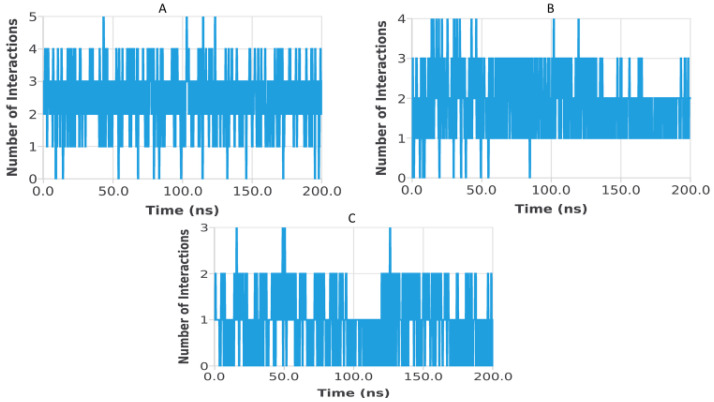
Hydrogen bond evolution of EGFR (5JRQ) complexes with ligands C5, C6, and C7 over 200 ns. (**A**) 5JRQ–C5, (**B**) 5JRQ–C6, (**C**) 5JRQ–C7.

**Figure 24:**
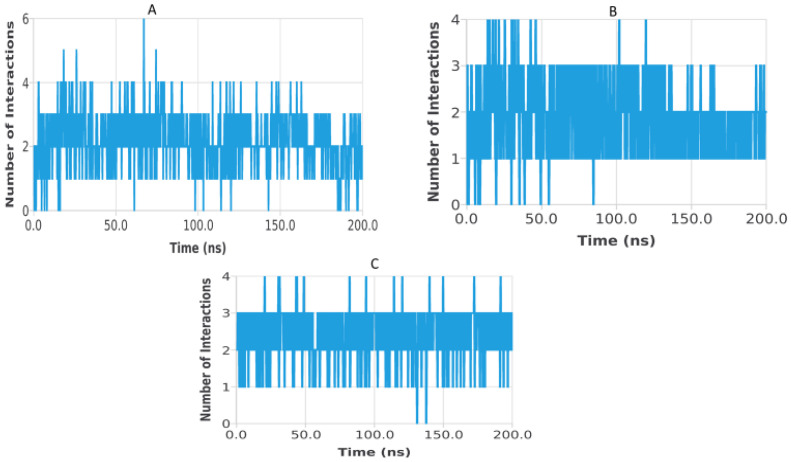
Hydrogen bond evolution of BRAF kinase (1M17) complexes with ligands C5, C6, and C7 over 200 ns. (**A**) 1M17–C5, (**B**) 1M17–C6, (**C**) 1M17–C7.

Hydrogen bond analysis was performed to assess the persistence and strength of polar interactions formed between ligands C5, C6, and C7 and each of the three target proteins during the 200 ns molecular dynamics simulations. This parameter provides essential insights into the dynamic stability of the ligand–protein interactions and complements structural descriptors such as RMSD, RMSF, Rg, SASA, and PSA. For the TGF-β receptor (3TZM), the hydrogen bond profiles shown in Figure 22 reveal distinct interaction patterns for the three ligands. Ligand C7 forms the most continuous and persistent hydrogen bonds, frequently maintaining 2–4 interactions over extended segments of the trajectory. Ligand C5 exhibits moderate but recurrent interactions, typically ranging from 1 to 3 hydrogen bonds, while C6 shows fewer and more intermittent contacts, fluctuating mostly between 0 and 2 bonds. These profiles demonstrate that all ligands remain stably anchored within the binding pocket, with C7 forming the strongest polar interaction network. For the EGFR (5JRQ), the hydrogen bond evolution illustrated in Figure 23 indicates that ligand C5 forms the highest number of hydrogen bonds, occasionally reaching 3–4 interactions and maintaining recurrent contacts throughout the 200 ns simulation. Ligand C7 exhibits a more uniform and stable profile, generally remaining between 1 and 3 hydrogen bonds, suggesting a consistent polar anchoring within the catalytic cavity. Ligand C6 shows fewer but regularly recurring hydrogen bonds, fluctuating between 0 and 3 interactions, suggesting that while its polar contacts are less continuous, they remain sufficient to support binding stability. For the BRAF kinase (1M17), the hydrogen bond patterns presented in Figure 24 show lower hydrogen bond counts overall compared with the other two proteins. Ligand C6 displays the most stable profile, maintaining intermittent but repetitive interactions that contribute to its binding persistence. Ligands C5 and C7 exhibit more variable patterns, generally oscillating between 0 and 1 hydrogen bond, yet they consistently maintain polar contacts with key residues throughout the simulation. Overall, the comparative hydrogen bond analysis confirms that each ligand maintains stable polar interactions with its respective protein target over the 200 ns simulation. Among the three compounds, C7 shows the strongest and most persistent hydrogen bonding pattern with TGF-β, C5 forms the highest number of hydrogen bonds with EGFR, and C6 exhibits the most regular hydrogen bond behavior with BRAF. These findings support the molecular dynamics results and highlight the complementary roles of polar and nonpolar interactions in stabilizing the ligand–protein complexes.

#### 2.4.3. MM-GBSA Energy Decomposition Analysis

MM-GBSA calculations were performed to estimate the binding free energy (ΔG bind) of the complexes formed by the cannabinoid ligands C5, C6, and C7 with the three cancer-related proteins EGFR, BRAF V600E, and TGF-β. This method integrates molecular mechanics energy terms and solvation contributions to provide an estimation of the thermodynamic stability of the examined protein–ligand complexes. The obtained ΔG bind values (Table 7) indicate favorable and stable binding affinities toward all three targets. TGF-β receptor: ΔG bind values were −58.78 kcal/mol for C5, −39.38 kcal/mol for C6, and −33.27 kcal/mol for C7. BRAF V600E: ΔG bind values were −56.81 kcal/mol for C5, −38.79 kcal/mol for C6, and −57.00 kcal/mol for C7. EGFR: ΔG bind values were −28.82 kcal/mol for C5, −38.56 kcal/mol for C6, and −57.30 kcal/mol for C7 [53]. These results confirm that the three cannabinoids form thermodynamically stable complexes with all examined protein targets, in agreement with the MD simulation analysis. TGF-β receptor: ΔG bind values were −67.45 kcal/mol for C5, −74.89 kcal/mol for C6, and −71.18 kcal/mol for C7 [54].

These results indicate that all three cannabinoids form stable complexes with each target, consistent with the MD analysis.

Table 8 presents the total binding free energies (ΔG bind) along with their decomposition into van der Waals (ΔG_vdw), electrostatic (ΔG_coul), hydrogen bonding (ΔG_hbond), nonpolar/lipophilic (ΔG_lip), and polar solvation (ΔG_solv) contributions for all ligand–protein complexes studied.

## 3. Discussion

These results indicate that these three cannabinoids may produce very stable complexes via strong hydrogen bonding, hydrophobic contact, and π–π interactions, beyond that which is presented by these reference drugs. The results indicate that the three selected cannabinoids (C5, C6, and C7) form highly stable complexes with EGFR, BRAF V600E, and TGF-β through strong hydrogen bonding, hydrophobic contacts, π–π interactions, and van der Waals forces. The interaction patterns suggest that C7 consistently exhibits the most favorable binding profile, which may explain its higher binding energies observed in docking studies. Although other compounds such as C9 and C11 showed promising docking scores, ADMET profiling allowed for prioritization of ligands with optimal pharmacokinetic and safety profiles. Overall, these findings support the potential of these cannabinoids as multitarget modulators, interfering with key signaling pathways involved in skin cancer progression and representing promising leads for dermotherapeutic and anti-skin cancer applications [55]. The physicochemical properties of these compounds suggest a favorable balance between hydrophilicity and lipophilicity, supporting effective skin permeability and bioavailability [56]. LogP values ranging from 1.60 to 4.05 indicate adequate lipophilicity, crucial for penetration through the lipid-rich layers of the skin. As summarized in Table 6, Table 7 and Table 8, all three compounds comply with Lipinski’s and Veber’s rules, exhibiting molecular weights below 500 g/mol, moderate to high lipophilicity, limited hydrogen bond donors and acceptors, and optimal rotatable bonds and polar surface area. These features ensure good permeation through the stratum corneum and subsequent bioavailability in the viable epidermis or dermis. High gastrointestinal absorption is predicted for all compounds, reflecting overall good systemic bioavailability; for topical applications, this primarily ensures safety in case of minor systemic exposure [57]. The calculated skin permeability coefficients (Log Kp between −2.737 and −2.538 cm/s) indicate moderate transdermal penetration, allowing delivery of bioactive molecules to target skin layers without excessive systemic absorption. These properties suggest that C5–C7 are excellent candidates for dermocosmetic applications, including antioxidant, anti-inflammatory, or anti-aging formulations. The ADME-Tox analysis further confirmed the safety and metabolic compatibility of these compounds. None of them are predicted to inhibit major CYP450 isoforms (CYP1A2, CYP2C19, CYP2C9, CYP2D6, CYP3A4), reducing the risk of metabolic interactions. Toxicity assessment via ProTox-II indicated that all three compounds are non-hepatotoxic, non-carcinogenic, non-mutagenic, and non-cytotoxic, with LD50 values ranging from 482 to 13,500 mg/kg, supporting a high safety margin for topical use. BBB predictions showed low permeation for C5 and C7, with controlled permeability for C6, minimizing potential CNS-related side effects [58]. The stability of the ligand–protein complexes was further confirmed by MM-GBSA calculations, which showed highly negative ΔG bind values, indicating thermodynamically favorable and strong interactions with EGFR, BRAF V600E, and TGF-β. Among the three ligands, C6 exhibited the most negative binding energies, suggesting slightly higher stability and affinity across all targets. These results, together with docking and MD simulation outcomes, reinforce the multi-target potential of these cannabinoids. Post-simulation protein–ligand interaction analyses were conducted to evaluate the persistence of key interactions observed during molecular docking. Hydrogen bond interactions were found to be limited and occurred only in a subset of complexes. Specifically, C5 formed a stable hydrogen bond with Ser280 in the 3TZM complex and with His539 in the 5JRQ complex, while C7 exhibited a hydrogen bond with Val219 in the 5JRQ complex. These interactions were consistent with those identified during molecular docking, indicating a high level of agreement between static docking predictions and dynamic simulation results (Figure 1, Figure 2 and Figure 3). In terms of interaction energetics, the overall stability of the cannabinoid–protein complexes appear to be predominantly governed by van der Waals forces and electrostatic contributions, as reflected in the MM-GBSA decomposition profiles. Among the three ligands, C7 shows the most favorable binding free energies toward EGFR and BRAF V600E (−57.30 and −57.00 kcal/mol, respectively), while C5 displays the strongest affinity toward the TGF-β receptor (−58.78 kcal/mol). These values suggest that both C5 and C7 achieve highly stable interactions within their respective binding pockets. The decomposition analysis indicates that van der Waals terms and Coulombic interactions represent the major stabilizing components of the binding process, whereas polar solvation effects tend to counteract these contributions to some extent. C6 exhibits moderate binding across all targets, with ΔG bind values ranging from −38.56 to −39.38 kcal/mol, reflecting weaker overall stabilization compared to C5 and C7. Differences in hydrogen-bonding contributions and lipophilic interactions further account for the variability observed among the three ligands. Overall, these energetic patterns highlight the importance of hydrophobic packing and electrostatic complementarity in driving stable cannabinoid binding to the EGFR, BRAF V600E, and TGF-β targets. Overall, the decomposition analysis provides detailed insights into the energetic factors governing ligand–protein recognition, highlighting the importance of hydrophobic and electrostatic contacts in stabilizing these complexes.

The strong binding and stability within the active sites imply that these compounds may effectively modulate key signaling pathways associated with cancer progression, highlighting their promise as leads for dermotherapeutic and anti-skin cancer agents [59].

## 4. Materials and Methods

### 4.1. Target Selection

The selection of molecular targets for this study focused on three key signaling pathways involved in skin cancer progression: EGFR (Epidermal Growth Factor Receptor), BRAF V600E (B-Raf proto-oncogene), and TGF-β (Transforming Growth Factor-beta). These targets were chosen based on their well-established roles in oncogenesis and tumor progression:

EGFR is overexpressed in multiple skin cancers, contributing to uncontrolled cell proliferation, migration, and survival [60].

BRAF V600E, particularly this V600E mutation, is a driver of melanomagenesis and has been implicated in aggressive skin cancer phenotypes [61].

TGF-β plays a dual role in tumor biology, acting as a tumor suppressor in early stages and as a promoter of invasion, metastasis, and fibrosis in advanced stages of skin cancer [27].

*Cannabis sativa*-derived phytoconstituents were selected as candidate modulators of these pathways due to their reported antiproliferative, anti-inflammatory, and pro-apoptotic properties in various cancer models. Computational screening of these bioactive compounds against EGFR, BRAF V600E, and TGF-β aims to identify potential multi-target inhibitors that may contribute to the development of novel therapeutic strategies for skin oncology.

### 4.2. Dataset of Candidate Compounds

Phytoconstituents were retrieved from a curated *Cannabis sativa* database, encompassing structurally characterized cannabinoids and terpenes reported for anticancer activity [62]. Selection criteria included chemical diversity, literature-supported bioactivity, and availability of high-quality 3D structures suitable for molecular docking studies. This approach ensured a representative and reliable set of compounds for computational screening against the selected oncogenic targets [63].

**Figure 25:**
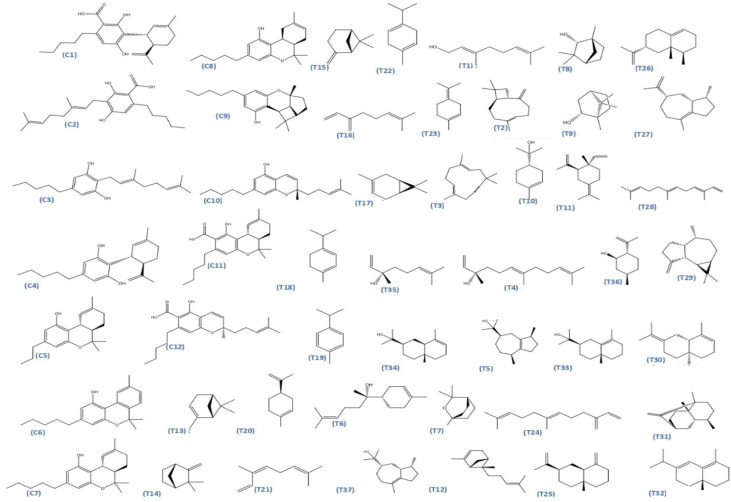
Chemical structures of the studied *Cannabis sativa* phytoconstituents.

The dataset used in this study consists of 49 phytochemicals derived from *Cannabis sativa*, selected for their documented therapeutic relevance and structural diversity. This dataset has been employed in previous publications from our laboratory [64]. Ensuring consistency and reproducibility in our analyses. Three-dimensional structures of all compounds were obtained from the PubChem database and subsequently geometry-optimized and energy-minimized under the MMFF94 force field using the steepest descent algorithm in Avogadro. Optimized structures were saved as PDB files and converted to PDBQT format using AutoDock Tools, making them ready for molecular docking. The dataset includes major and minor cannabinoids (e.g., Δ^9^-THC, CBD, CBG, THCV) as well as representative terpenes commonly found in cannabis resin. The 2D structures and PubChem Compound Identifiers (CIDs) are provided in Table 9 and Figure 25.

Figure 25 presents the chemical structures of 49 *Cannabis sativa* phytoconstituents, including cannabinoids (C1–C12) and terpenes (T1–T37), used in this study. Each compound is labeled with its corresponding number, as referenced throughout the manuscript in docking, molecular dynamics, and interaction analyses. The figure provides a visual overview of all tested phytoconstituents, facilitating identification and cross-referencing with Table 8 and associated abbreviations listed below.

### 4.3. Molecular Docking

To investigate the potential anticancer activity of *Cannabis sativa* phytoconstituents, molecular docking studies were conducted against three major oncogenic targets involved in skin cancer progression: epidermal growth factor receptor (EGFR), BRAF V600E mutant kinase, and transforming growth factor-beta (TGF-β) [65]. These targets are critically implicated in tumor proliferation, migration, invasion, and resistance to therapy, making them ideal candidates for a multitarget drug discovery approach. Docking simulations were performed using Auto Dock Vina 1.1.2 (The Scripps Research Institute, La Jolla, CA, USA, 2010) [66], which employs a semi-flexible docking protocol maintaining the protein targets as rigid while allowing full conformational flexibility of the ligands. Default parameters were applied, including an exhaustiveness value of 8, an energy range of 4, and the generation of 9 binding poses per compound [67]. The 3D crystal structures of EGFR, BRAF V600E, and TGF-β were retrieved from the RCSB Protein Data Bank (https://www.rcsb.org, last accessed 12 December 2025) and prepared using Auto Dock Tools v1.5.6 (The Scripps Research Institute, La Jolla, CA, USA, 2001). Protein preparation involved the removal of crystallographic water molecules, addition of polar hydrogens, and assignment of Gasteiger charges. All selected phytoconstituents were geometry-optimized and converted into the appropriate PDBQT format following energy minimization [68]. To benchmark the performance of the tested phytoconstituents, Erlotinib, an FDA-approved tyrosine kinase inhibitor targeting EGFR, was docked against all three targets to provide a benchmark for evaluating the relative performance of the tested cannabis-derived molecules. The binding affinities, modes of interaction, and involved residues were analyzed and visualized using Discovery Studio Visualizer 2021 (Dassault Systems, San Diego, CA, USA, 2021) [69], with special focus on hydrogen bonds, hydrophobic interactions, and key active site contacts for each target.

#### 4.3.1. Ligand Preparation

The three-dimensional (3D) structures of forty-nine selected phytoconstituents from *Cannabis sativa*, were retrieved from the PubChem database (National Center for Biotechnology Information, Bethesda, MD, USA). These compounds, mainly cannabinoids and terpenes, were selected based on their documented bioactivity and structural diversity. Prior to docking, the ligands were geometry-optimized using the MMFF94 force field and the Steepest Descent algorithm implemented in Avogadro software (Open Chemistry, Calgary, AB, Canada, 2020) [70], to ensure low-energy conformations compatible with protein interaction. The optimized molecules were then converted into PDBQT format using AutoDock Tools v1.5.6, in preparation for molecular docking simulations.

#### 4.3.2. Protein Preparation

Crystal structures of target proteins associated with skin cancer—BRAF V600E, EGFR, and TGF-β receptors—were downloaded from the RCSB Protein Data Bank in PDB format and prepared using BIOVIA Discovery Studio 2021 (Dassault Systems, San Diego, CA, USA, 2021). Preprocessing included the removal of water molecules and heteroatoms, addition of polar hydrogen atoms, and charge assignment using Kollman and Gasteiger. The prepared files were saved in PDBQT format for docking in AutoDock Vina. Grid box parameters were optimized carefully to include active sites for all the target proteins to allow for proper calculation of ligand–protein interactions [71]. Detailed coordinates and dimensions of the grid boxes for each protein are provided in Table 10.

**Figure 26:**
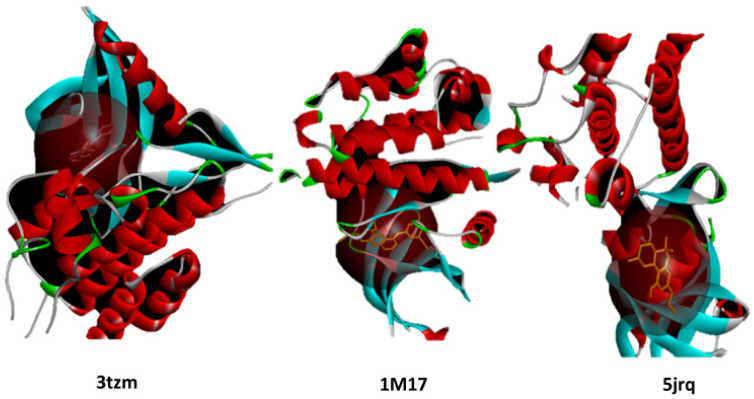
Visualization of the active site grid box positioning for EGFR, BRAF V600E, and TGF-β proteins based on their co-crystallized ligands. The grid boxes define the docking regions used in the molecular docking simulations. 3tzm; 1m17; 5jrq.

To ensure accurate targeting of the biologically relevant binding sites, molecular docking simulations were performed using grid boxes centered on the active sites of each protein, as defined by their respective co-crystallized ligands. The grid box dimensions were selected to fully encompass the binding pocket and key interacting residues while allowing sufficient conformational flexibility for ligand accommodation. This approach ensures that all docked phytoconstituents explore the same functional region responsible for ligand recognition and biological activity (Figure 26).

### 4.4. Pharmacokinetics and Drug-Likeness Prediction (ADMET Analysis)

Pharmacokinetic and toxicity profiles were predicted using SwissADME (Swiss Institute of Bioinformatics, Lausanne, Switzerland) [72], ProTox-II (Charité—Universitätsmedizin Berlin, Berlin, Germany) [73], and pkCSM [74]. Canonical SMILES of each compound were submitted to these platforms to predict absorption, distribution, metabolism, excretion, and toxicity-related parameters relevant to dermal applications. The predicted ADME-Tox parameters of C5, C6, and C7 are summarized in Table 4, Table 5 and Table 6. These in silico ADME-Tox evaluations provide a rapid and cost-effective preliminary screening strategy, reducing experimental workload and prioritizing the most promising candidates for subsequent in vitro and in vivo validation [8].

### 4.5. Molecular Dynamics Simulation

In this study, we evaluated the stability of the selected multi-target ligands against EGFR, BRAF V600E, and TGF-β using Schrödinger Maestro (Schrödinger Inc., New York, NY, USA, 2021) for 100 ns [75,76]. Protein structures were prepared by removing overlapping water molecules and solvating the systems with TIP3P water. To maintain neutrality and physiological ionic strength, Na^+^ and Cl^−^ ions were added to achieve a salt concentration of 0.15 M. The OPLS3e force field was applied to parameterize the proteins [77,78]. Temperature and pressure were maintained using an isothermal–isobaric ensemble (NPT) with the Nose–Hoover thermostat and Martyna–Tobias–Klein barostat, following standard MD protocols [79]. Equilibration was performed in two stages: a 1 ns NVT phase, followed by NPT equilibration at 310.5 K and 1.01 bar, before running the production simulation for 100 ns. Trajectories were recorded every 100 ps, generating approximately 1000 frames. Post-simulation analysis included root mean square deviation (RMSD), root mean square fluctuation (RMSF), radius of gyration (Rg), solvent-accessible surface area (SASA), and protein–ligand contact analyses to evaluate structural stability, flexibility, and interaction dynamics throughout the simulation.

This MD study provided critical insights into the time-dependent behavior and binding persistence of the selected *Cannabis sativa* cannabinoids within their respective target binding sites, supporting their potential role as multi-target modulators in skin cancer therapy [80].

### 4.6. MM-GBSA Calculations

The binding free energies (ΔG bind) of the selected protein–ligand complexes were calculated using the MM-GBSA approach in Schrödinger Maestro (Schrödinger Inc., New York, NY, USA, 2021). The Prime MM-GBSA module was used to perform the calculations, preparing the protein–ligand complex and retaining it in the workspace as per the project table. This involved the application of the VSGB solvation model along with the OPLS3e force field to calculate the free-energy estimations precisely and reliably. This workflow enabled the quantitative assessment of binding affinities for the selected cannabinoid ligands toward the three investigated targets [81].

The MM-GBSA binding free energy calculations were carried out using snapshots extracted from the equilibrated region of the 200 ns molecular dynamics trajectories. A total of 1000 frames, saved at 100 ps intervals, were considered for each protein–ligand complex to ensure robust statistical sampling. The total binding free energy (ΔG bind) was decomposed into van der Waals, electrostatic, polar solvation, and non-polar solvation contributions [82].

While the current study is computational, it provides a prioritized list of Cannabis-derived compounds for experimental validation. Future work should include cytotoxicity assays, pathway-specific expression analyses (Western blot or RT-PCR), and functional assays for apoptosis, migration, and oxidative stress in skin cancer cell lines. These experiments will be essential to confirm the mechanistic predictions suggested by our in-silico analyses. However, the computational results suggest that the shortlisted compounds may interact with and potentially modulate the TGF-β signaling pathway. This is supported by high docking scores, stable molecular dynamics interactions with key residues, and literature-reported activities of structurally similar phytochemicals. However, these predictions remain computational, and experimental validation is required to confirm their biological effect.

## 5. Conclusions

This study comprehensively investigated the potential of *Cannabis sativa* phytoconstituents as putative multi-target inhibitors of key proteins implicated in skin cancer, namely EGFR, BRAF V600E, and TGF-β, by integrating molecular docking, ADMET profiling, molecular dynamics simulations, and MM-GBSA binding energy analysis. Among the forty-nine compounds evaluated, three phytochemicals—C5, C6, and C7—consistently demonstrated the highest binding affinities, forming stable and specific interactions within the active sites of all three targets. These ligands displayed strong complementarity to the binding pockets, high thermodynamic stability, and favorable molecular interactions, supporting their potential as multi-target modulators of oncogenic signaling pathways. The ADMET analysis revealed that C5, C6, and C7 possess promising pharmacokinetic properties, including good predicted oral bioavailability and minimal toxicity, with no predicted mutagenic, hepatotoxic, or skin-sensitizing effects. Molecular dynamics simulations further supported these findings, confirming the structural stability and compactness of the ligand–protein complexes under biomimetic conditions.

Taken together, these computational results highlight the value of an integrated in silico framework for identifying and prioritizing candidate compounds in early-stage drug discovery. C5, C6, and C7 emerge as computationally validated leads worthy of further experimental investigation. While in vitro and in vivo studies remain essential to confirm their biological activity and therapeutic potential, this work provides a rational foundation for future research into multi-target phytochemical-based strategies for skin cancer treatment.

## Figures and Tables

**Table 1:** Binding free energies (ΔG, kcal/mol) of docked *Cannabis sativa* phytoconstituents against EGFR, BRAF V600E, and TGF-β.

Ligand	BE (Kcal/mol)			Ligand	BE (Kcal/mol)			Ligand	BE (Kcal/mol)		
	EGFR	BRAF	TGF		EGFR	BRAF	TGF		EGFR	BRAF	TGF
C1	−7.4	−6.9	−8.6	T6	−5.2	−6.4	−6.4				
C2	−7.1	−6.5	−7.8	T7	−5.2	−6.6	−5.1	T23	−6.5	−6.4	−5.5
C3	−6.8	−6.3	−7.3	T8	−6.2	−6.2	−5.5	T24	−6.1	−6.4	−5.3
C4	−7.3	−7.4	−8.1	T9	−6.6	−5.7	−5.3	T25	−6.1	−5.9	−5.0
C5	−8.5	−9.9	−9.4	T10	−6.3	−6.6	−5.0	T26	−5.1	−6.0	−5.6
C6	−8.8	−9.6	−9.2	T11	−6.3	−6.5	−5.6	T27	−6.4	−6.6	−5.4
C7	−9.6	−9.8	−9.2	T12	−5.5	−5.6	−5.4	T28	−6.5	−5.1	−5.3
C8	−8.9	−7.1	−8.6	T13	−5.7	−6.2	−5.3	T29	−6.6	−5.5	−5.5
C9	−8.0	−8.1	−8.7	T14	−5.0	−6.2	−5.5	T30	−7.6	−5.3	−6.0
C10	−7.8	−7.0	−8.6	T15	−6.0	−5.4	−6.6	T31	−5.6	−5.0	−6.6
C11	−8.5	−7.6	−8.5	T16	−5.7	−6.8	−5.2	T32	−6.7	−5.6	−5.1
C12	−8.0	−6.6	−8.4	T17	−6.0	−5.0	−5.1	T33	−6.8	−5.4	−5.5
T1	−6.9	−5.2	−5.4	T18	−7.0	−5.5	−5.2	T34	−5.6	−5.3	−5.3
T2	−6.6	−5.1	−5.5	T19	−6.4	−5.8	−5.7	T35	−5.5	−5.5	−5.3
T3	−6.9	−5.2	−5.3	T20	−5.9	−6.3	−5.3	T36	−5.8	−5.7	−5.5
T4	−6.6	−5.7	−5.7	T21	−6.1	−6.5	−5.6	T37	−5.9	−5.6	−6.6
T5	−6.2	−5.3	−5.6	T22	−6.5	−6.0	−5.5	T38	−5.0	−5.5	−5.2
Reference drugs											
Erlotinib	−7.5	-	-	Vemurafenib	-	−6.5	-	Galunisertib	-	-	−6.1

BE, binding energy (kcal/mol); EGFR, epidermal growth factor receptor; BRAF V600E, v-raf murine sarcoma viral oncogene homolog B1; TGF-β, transforming growth factor beta.

**Table 2:** Top-ranking *Cannabis sativa* phytoconstituents (C5–C7) and reference drug binding affinities against EGFR, BRAF V600E, and TGF-β.

Ligand	Name	Binding Affinities kcal/mol		
		EGFR (PDB ID: 1M17)	BRAF (PDB ID: 5JRQ)	TGF-β (PDB ID: 3TZM)
C5	THCV	−9.4	−8.5	−7.7
C6	CNB	−9.2	−8.8	−7.4
C7	9-THC/Dronabinol	−9.2	−8.5	−7.5
Reference Drug	Erlotinib	−8.1	−7.6	−7.2

EGFR, epidermal growth factor receptor; BRAF V600E, B-Raf proto-oncogene serine/threonine kinase; TGF-β, transforming growth factor beta; THCV, tetrahydrocannabivarin; CNB, cannabinol; Δ^9^-THC, delta-9-tetrahydrocannabinol (dronabinol); PDB, Protein Data Bank; kcal/mol, kilocalories per mole.

**Table 3:** Interaction profiles of C5, C6, and C7 with 3TZM, 1M17, and 5JRQ.

Ligand	3tzm Residues	Interaction Types	Distance (å)	1m17 Residues	Interaction Types	Distance (å)	5jrq Residues	Interaction Types	Distance (å)
c5	ser280, tyr282, leu289, leu340, ile211	H-bond, π–π stacked, π–alkyl, hydrophobic	2.6–4.9	phe699, leu820, val702, ala719	π–π stacked, π–alkyl, hydrophobic	3.6–5.0	his539, lys473	H-bond, π–alkyl	3.2–4.8
c6	leu278, val219, ala230, tyr249	π–alkyl, π–σ, hydrophobic	3.5–5.2	asp831, phe699, leu820, val719	H-bond, π–π stacked, hydrophobic	2.4–5.2	lys473, leu471	π–alkyl, hydrophobic	3.6–4.0
c7	val219, leu260, ala230, ile211, tyr282	π–σ, π–alkyl, hydrophobic	3.4–4.9	ala719, leu820, lys721, phe699	π–alkyl, π–π stacked, π–cation	3.5–5.0	gln461, leu471, ile467	H-bond, π–alkyl, hydrophobic	2.7–4.9

H-bond: Conventional hydrogen bond, π–π stacked: Pi–pi stacking, π–alkyl: Pi–alkyl interaction, π–σ: Pi–sigma interaction, π–cation: Pi–cation interaction, Hydrophobic: Hydrophobic contact.

**Table 4:** Molecular properties and ADME prediction of C5–C7 compounds.

Ligand	MW (g/mol)	LogP	HBA	HBD	Rot N	Surface Area Å^2^	Solubility	LogS (mol/L)	Lipinski	Veber
C5	314.46	4.05	2	1	2	40.46	Moderately Soluble	−5.41	Yes	Yes
C6	314.46	1.60	2	1	2	40.46	Moderately Soluble	−5.74	Yes	Yes
C7	286.41	3.99	2	1	2	29.46	Poorly Soluble	−6.11	Yes	Yes

MW: Molecular Weight; LogP: Logarithm of the partition coefficient.

**Table 5:** ADMET and Skin Permeation of C5–C7 compounds.

Ligand	Bioavailability Score	PAINS Alerts	GI Absorption	BBB Permeant	CYP1A2	CYP2C19	CYP2C9	CYP2D6	CYP3A4	Log Kp Skin Permeation
C5	0.55	0	High	No	No	Yes	Yes	Yes	No	−2.737
C6	0.55	0	High	Yes	Yes	No	Yes	Yes	No	−2.538
C7	0.55	0	High	No	No	Yes	Yes	Yes	No	−2.538

PAINS, pan-assay interference compounds; GI, gastrointestinal absorption; BBB, blood–brain barrier; CYP1A2, cytochrome P450 1A2; CYP2C19, cytochrome P450 2C19; CYP2C9, cytochrome P450 2C9; CYP2D6, cytochrome P450 2D6; CYP3A4, cytochrome P450 3A4; Log Kp, logarithm of skin permeability coefficient.

**Table 6:** ProTox-II Toxicity Predictions of C5–C7 compounds.

Ligand	Hepatotoxicity	Carcinogenicity	Mutagenicity	Cytotoxicity	LD50 (mg/kg)	Class
C5	Inactive	Inactive	Inactive	Inactive	482	4
C6	Inactive	Inactive	Inactive	Inactive	13,500	6
C7	Inactive	Inactive	Inactive	Inactive	482	4

LD50, median lethal dose (mg/kg); Class, acute oral toxicity class according to the Globally Harmonized System (GHS).

**Table 7:** MM-GBSA binding free energies (ΔG bind, kcal/mol) of the three selected cannabinoids (C5, C6, C7) against the three cancer-related protein targets.

Ligand	MM-GBSA Binding Affinities (Kcal/mol)		
	EGFR	BRAF	TGF-β
C5	28.82	−56.81	−58.78
C6	−38.56	−38.79	−39.38
C7	−57.30	−57.00	−33.27

MM-GBSA, Molecular Mechanics/Generalized Born Surface Area; EGFR, epidermal growth factor receptor; BRAF, BRAF V600E, B-Raf proto-oncogene serine/threonine kinase; TGF-β, transforming growth factor beta.

**Table 8:** MM/GBSA binding free energies (kcal/mol) and energy decomposition for ligands C5–C7 with EGFR, BRAF V600E, and TGF-β.

Ligand	Protein	ΔG Bind	ΔG_vdw	ΔG_coul	ΔG_hbond	ΔG_lip	ΔG_solv
C5	EGFR	−28.82	150.311	−38,574.541	−299.860	−1406.775	−5897.190
C5	BRAF	−56.81	25.082	−74.255	0.00	−4.020	−3.617
C5	TGF-β	−58,775	21.988	−73.858	0.00	−3.773	−3.132
C6	EGFR	−38.56	−11.275	−17,716.469	−147.263	−688.365	−3888.909
C6	BRAF	−38,794	25.767	−63.811	0.00	−3.580	−4.330
C6	TGF-β	−39,382	32.095	−64.389	0.00	−3.706	−3.382
C7	EGFR	−57.30	152.592	−19,157.484	−131.609	−693.741	−2692.740
C7	BRAF	−57.00	−213.653	−12,508.120	−99.749	−556.860	−2243.786
C7	TGF-β	−33.27	−116.122	−13,730.472	−114.959	−527.049	−2195.587

ΔG bind, total MM-GBSA binding free energy; ΔG vdw, van der Waals interaction energy; ΔG_coul, electrostatic (Coulomb) interaction energy; ΔG hbond, hydrogen bonding contribution; ΔG_lip, lipophilic contribution; ΔG_solv, solvation free energy; EGFR, epidermal growth factor receptor; BRAF V600E, B-Raf proto-oncogene serine/threonine kinase; TGF-β, transforming growth factor beta.

**Table 9:** 2D structures and trivial names of selected phytoconstituents.

ID	Name	Structure	ID	Name	Structure
C1	CBDA	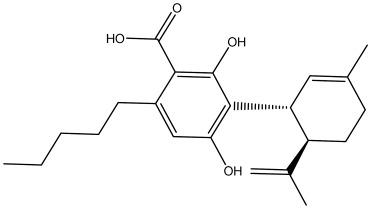	T1	Geraniol	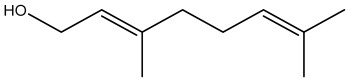
C2	CBGA	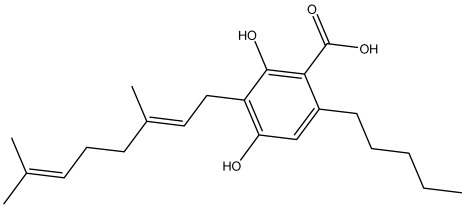	T2	β-Caryophyllene	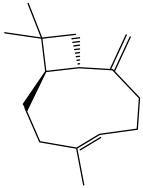
C3	CBG	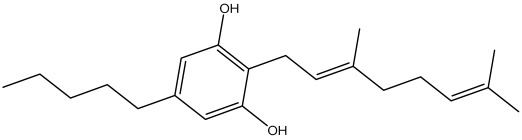	T3	α-Humulene	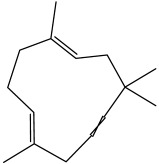
C4	CBD	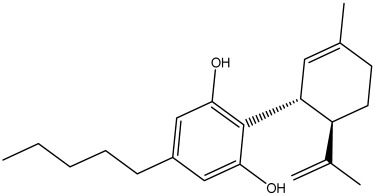	T4	Nerolidol	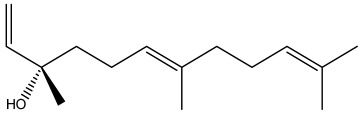
C5	THCV	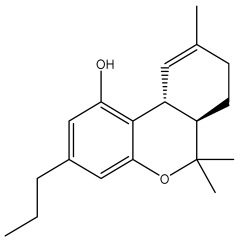	T5	(−)-Guaiol	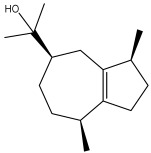
C6	CNB	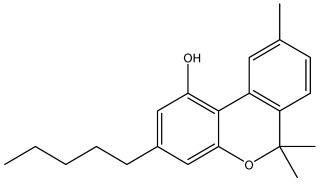	T6	(−)-α-Bisabolol	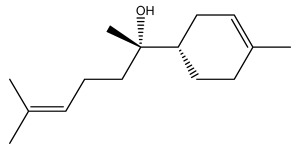
C7	Δ-9-THC	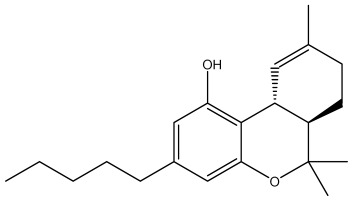	T7	Cineol	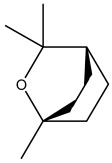
C8	Δ-8-THC	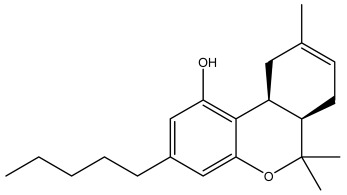	T8	Fenchol	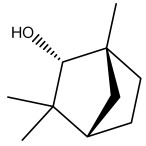
C9	CBL	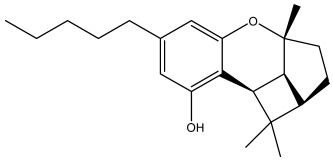	T9	Borneol	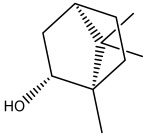
C10	CBC	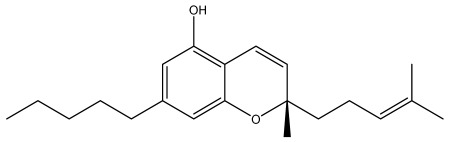	T10	α-Terpineol	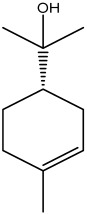
C11	THCA	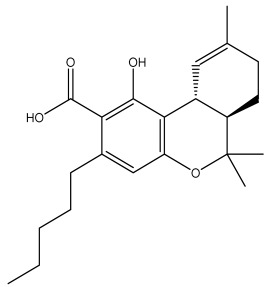	T11	γ-Elemene	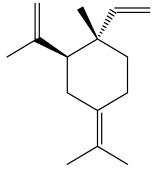
C12	CBCA	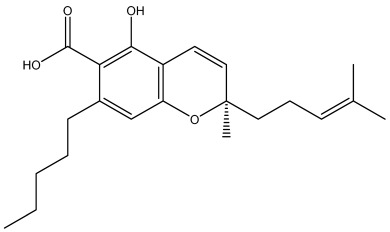	T12	α-Bergomotene	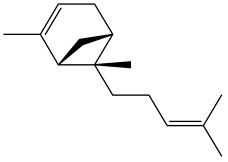
T13	α-Pinene	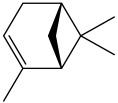	T24	β-Farnesene	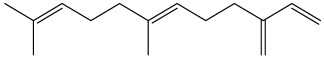
T14	Camphene	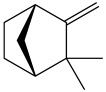	T25	β-Eudesmene	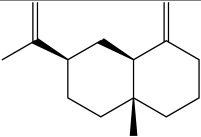
T15	β-Pinene	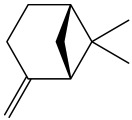	T26	Valencene	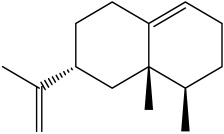
T16	β-Myrcene	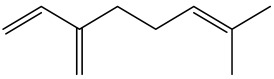	T27	α-Bulnesene	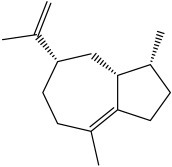
T17	δ-3-Carene	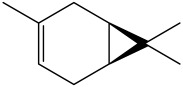	T28	Farnesene	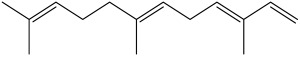
T18	α-Terpinene	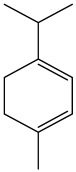	T29	β-Gurjunene	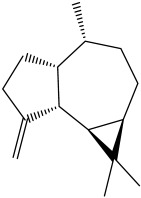
T19	p-Cymene	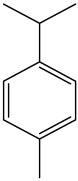	T30	Eudesma-3,7(11)-diene	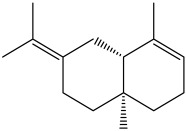
T20	d-Limonene	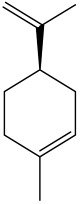	T31	Seychellene	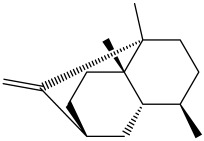
T21	Ocimene	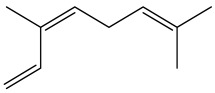	T32	δ-Selinene	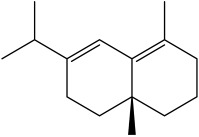
T22	γ-Terpinene	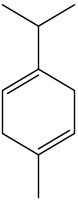	T33	γ-Eudesmol	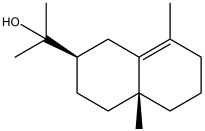
T23	Terpinolene	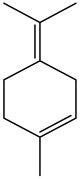	T34	α-Eudesmol	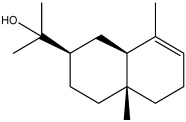
T35	Linalool	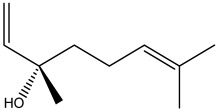	T37	Bulnesol	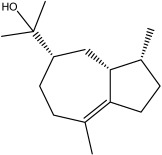
T36	(−)-Isopulegol	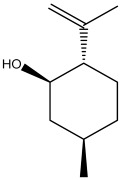			

CBDA: Cannabidiolic acid; CBGA: Cannabigerolic acid; CBG: Cannabigerol; CBD: Cannabidiol; THCV: Tetrahydrocannabivarin; CNB: Cannabinol; Δ^9^-THC: Δ^9^-Tetrahydrocannabinol; Δ^8^-THC: Δ^8^-Tetrahydrocannabinol; CBL: Cannabicyclol; CBC: Cannabichromene; THCA: Tetrahydrocannabinolic acid; CBCA: Cannabichromenic acid.

**Table 10:** Summary of the target proteins used in molecular docking studies of Cannabis phytoconstituents against skin cancer-related targets.

PDB ID	Target Protein	Center X (Å)	Center Y (Å)	Center Z (Å)	Grid Box Size (Å) X × Y × Z
5JRQ	BRAF kinase	−11.294	−4.229	−29.292	40 × 40 × 40
1M17	EGFR tyrosine kinase	22.014	0.253	52.795	40 × 40 × 40
3TZM	TGF-β receptor	4.528	8.718	6.785	20 × 20 × 20

## Data Availability

The data presented in this study are available within the article. The raw computational data supporting the findings of this study, including docking results, ADMET predictions, MD trajectories, and MM-GBSA calculations, are available from the corresponding author upon reasonable request.
